# Unsupervised Monocular Visual Odometry for Fast-Moving Scenes Based on Optical Flow Network with Feature Point Matching Constraint

**DOI:** 10.3390/s22249647

**Published:** 2022-12-09

**Authors:** Yuji Zhuang, Xiaoyan Jiang, Yongbin Gao, Zhijun Fang, Hamido Fujita

**Affiliations:** 1 School of Electronic and Electrical Engineering, Shanghai University of Engineering Science, Shanghai 201600, China; 2 Faculty of Information Technology, HUTECH University, Ho Chi Minh City, Vietnam; 3 i-SOMET Inc., Morioka 020-0104, Japan; 4 Regional Research Center, Iwate Prefectural University, Takizawa 020-0693, Japan

**Keywords:** visual odometry, flow network, feature point matching, depth network, trajectory drift, SLAM

## Abstract

Robust and accurate visual feature tracking is essential for good pose estimation in visual odometry. However, in fast-moving scenes, feature point extraction and matching are unstable because of blurred images and large image disparity. In this paper, we propose an unsupervised monocular visual odometry framework based on a fusion of features extracted from two sources, that is, the optical flow network and the traditional point feature extractor. In the training process, point features are generated for scene images and the outliers of matched point pairs are filtered by FlannMatch. Meanwhile, the optical flow network constrained by the principle of forward–backward flow consistency is used to select another group of corresponding point pairs. The Euclidean distance between the matching points found by FlannMatch and the corresponding point pairs by the flow network is added to the loss function of the flow network. Compared with SURF, the trained flow network shows more robust performance in complicated fast-motion scenarios. Furthermore, we propose the AvgFlow estimation module, which selects one group of the matched point pairs generated by the two methods according to the scene motion. The camera pose is then recovered by Perspective-n-Point (PnP) or the epipolar geometry. Experiments conducted on the KITTI Odometry dataset verify the effectiveness of the trajectory estimation of our approach, especially in fast-moving scenarios.

## 1. Introduction

Simultaneous localization and mapping (SLAM) [1] is a core part of autonomous navigation systems. For example, robots can adopt SLAM to realize their localization and reconstruct the scene maps in unknown environments. Compared with SLAM systems, visual odometry (VO) focuses on the egomotion estimation of the agent itself, predicting the camera trajectory frame by frame using efficient features. In most cases, VO estimates the egomotion faster and more efficiently than SLAM systems. VO estimates the pose changing of the camera from adjacent frames. The estimated subsequent pose is based on the previous results, followed by an online local optimization process. Inevitably, trajectory drift accumulates as time goes on, which always leads to the failure of the VO system. Hence, robust and accurate visual feature tracking is essential for good pose estimation in visual odometry. Famous feature point extractors, such as, SIFT [2] and SURF [3], which is faster than SIFT, are the basis for accurate feature matching.

To reduce the accumulative error, researchers adopt the loop detection. The normal way to realize loopback detection is to perform a feature matching on any two images and determine whether two images are associated or not by the number of correctly matched feature points. ORB-SLAM2 [4] performs feature matching on the current frame and the loop candidate frame by adding a loop back detection module.

**Figure 1:**
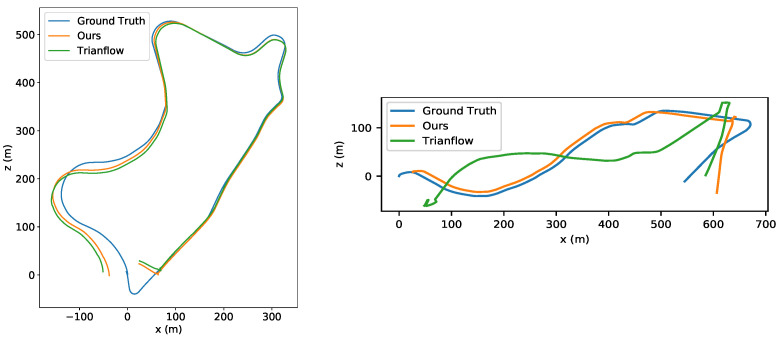
The estimated trajectories of sequences 09 and 10 from the KITTI Odometry dataset. We took the stride as four, that is, only one in every four frames was used for trajectory computing, to simulate fast moving. As shown, our model still worked well in fast-moving scenes.

However, in fast-moving scenes, the trajectory drift of traditional VO systems is more obvious than in normal cases. To improve the robustness of VO systems in fast-moving scenes, researchers began to adopt deep learning methods. Bian et al. [5] trained a depth network and pose network to ensure the consistency in long series prediction by geometric consistency constraints. They adopted the powerful feature extraction ability of neural networks to train a large number of datasets and estimate the camera pose by a series of video streams end to end. TrainFlow [6] is a self-supervised framework which combines an optical flow network and a depth network. The scale of depth estimation is added to the triangulated point cloud and the depth error is calculated using the converted depth map. Although they greatly improved the robustness of VO in fast motion scenes, the trajectory drift still exists, as shown in Figure 1.

In this paper, we propose an unsupervised monocular visual odometry based on an optical flow network with a feature point matching constraint. In the training process, the matched point pairs generated by the traditional feature matching method is assumed as the ground truth and is adopted as a constraint to train the optical flow network in an unsupervised manner. When the motion in the scenario is fast, the matching of feature points by traditional extractors are not reliable, causing the performance to decline and losing the pose estimation of the visual odometry systems. In contrast, points selected by the forward–backward flow consistency selection method are more accurate. Therefore, we present an adaptive feature matching selection module, that is, the AvgFlow estimation module, to select one of the corresponding point pairs generated by the two methods in different moving-speed situations. Finally, we recover the camera pose by the epipolar-geometry-based and PnP-based methods.

Our contributions are summarized as follows:

- We propose an unsupervised visual odometry framework that uses the matched feature points as the supervisory label for the training of the flow network.
- We present an adaptive feature matching selection module to obtain robust pose estimation performance in different motion scenarios, especially in fast-moving scenes.
- Experiments on the KITTI Odometry dataset show a significant accuracy improvement of the proposed approach compared with the traditional and the deep-learning-based visual odometry methods.

In this work, we propose a robust VO system for fast-moving scenes, incorporating traditional matching, named TFD-VO. The rest of the paper consists of the following sections: Section 2 mainly analyze the related works. Section 3 focuses on the proposed TFD-VO framework and the loss function. Section 4 shows the experimental results and evaluates the impact of different factors on the whole system through detailed ablation studies. Section 5 gives some conclusions.

## 2. Related Work

### 2.1. Traditional Visual Odometry

Traditional visual odometry mainly uses multiview geometry [7,8] theory to localize the camera based on the image information taken by the camera. The first pioneering traditional VO framework, parallel tracking and mapping (PTAM), was proposed by Klein et al. [9]. Although the performance was not perfect, it provided a complete and general framework. Later, Mur-Artal et al. [4] proposed ORB-SLAM2 based on that framework, and the initialization phase used an automatic mechanism to compute and score both homograph and fundamental matrices by matching oriented fast and rotated BRIEF (ORB) [10] features, without assuming whether the scene was planar or not.

However, the feature-based method discards most of the potentially useful image information, and the camera may move to places where feature points are missing, which often have no obvious texture information. So Engel et al. [11] proposed a direct sparse odometry (DSO) based on the direct method, which mainly sampled key points uniformly from the global image and added a complete photometric calibration, selecting key frames by judging the changes of exposure parameters between two frames. However, the direct method required a high level of scene lighting and needed to keep the exposure time stable. Therefore, Zhou et al. [12] proposed a new method to parameterize 3D collinear points; a 3D point was mainly determined by the inverse depth of the two endpoints of a 2D line. It added line features to the constraints without increasing the number of variables and the difficulty of the optimization.

To further improve the robustness of the VO system, Tian et al. [13] proposed a GPE algorithm based on Delaunay triangulation. It mainly used several geometric constraints to select and refine the feature points close to the ground and used RANSAC-based method to optimize the point set. After that, Company-Corcoles et al. [14] proposed a multiview Manhattan axis estimation method. It mainly searched for parallel or vertical line correspondence in local maps and selected redundant features. However, all these methods mentioned above have trajectory drift problems in fast-motion scene, which our proposed method can solve.

### 2.2. Deep-Learning-Based Visual Odometry

Deep learning has led to a number of achievements in computer-vision-related tasks. For instance, Zheng et al. [15] used deep learning to optimize the feature boundary of a deep CNN to reduce the overfitting problem. Zhao et al. [16] proposed a novel faster mean-shift algorithm using deep learning and achieved the highest computational speed. Yao et al. [17] used deep learning to propose a simple compound figure separation framework that could be efficiently deployed to new image classes without the need for extensive resource bounding box annotations. Jin et al. [18] used deep learning to propose a pseudo RGB-D face recognition framework, which replaced the depth sensor with a generated pseudodepth map.

Traditional visual odometry tends to lose tracking targets when the camera is moving too fast and is not very robust in some extreme scenes. To solve the problems, more and more visual odometry methods adopt deep learning models. However, supervised learning methods require large manual labeled datasets, which are often costly and difficult to obtain. Therefore, more and more people are focusing on unsupervised learning.

Zhou et al. [19] jointly trained a depth network and a pose network by minimizing the photometric error as the monitoring signal. Zhan et al. [20] found the connection of corresponding pixels between the front and back frames by a reconstruction of left and right views. They added the edge smoothness constraint to train the depth and pose networks. Bian et al. [5] proposed a geometric consistency constraint to ensure the scale consistency of depth and pose estimation networks in the prediction of long video sequence at the same time. Ranjan et al. [21] divided the picture into a moving part and static part through motion segmentation network and combined depth information, camera motion and optical flow to constrain and enhance the relationship between them. Li et al. [22] used Bayesian depth filters to improve data and used the method from Teed et al. [23] to learn the dense optical flow between the key frame and the current frame. Wang et al. [24] incorporated the inherent parameters of the camera into the model and proposed a new up-to-scale loss function to recover camera motion. Subsequently, Kuo et al. [25] built on the latter model by using flow maps to dynamically adjust the attention weights for different semantic categories, weighting the generated attention maps to pose estimation. To further improve the accuracy of the trajectory, Wang et al. [26] added motion constraints to the network and automatically adjusted the weights of the origin loss and the motion loss by the gradient descent rate.

However, the pose estimation of all the above methods is based on a CNN, whose scale is difficult to obtain. It is challenging for CNN-based pose estimators to accurately predict the pose in fast-motion scenes. To solve the problem, Zhao et al. [6] took the depth error between the triangulated depth and the depth predicted by the depth network as the self-supervised signal. Instead of a CNN-based pose estimation, they recovered the camera pose directly from the optical flow correspondence. Although the approach of Zhao et al. [6] achieved great results in fast-motion scenes, the trajectory drift phenomenon still existed. Due to the vehicle not always traveling at a constant speed, it still decelerates when it encounters a traffic light, and in some scenes, the vehicle may suddenly accelerate. As a result, the robustness of the method proposed by Zhao et al. [6] to recover the camera pose only from the optical flow correspondence is not very strong.

After investigating previous works, we found that the traditional matching method worked well in slow motion scenes but always lost tracks in fast-motion scenes. In contrast, a flow network has better performance in fast-moving scenarios. Therefore, we fused the traditional matching method and optical flow consistency to adaptively switch between optical flow and traditional matching method by judging how fast or slow the motion is between adjacent frames. Experiments show that our VO system is more robust.

## 3. The Proposed Approach

### 3.1. System Overview

**Figure 2:**
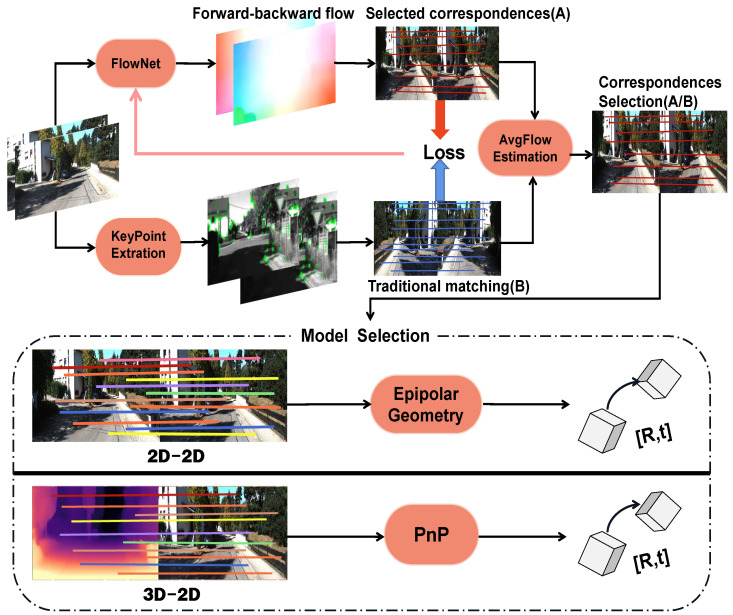
System overview. The FlowNet predicts optical flows between adjacent frames. Afterward, we use the forward–backward flow consistency score map to select the corresponding point pairs set, denoted as A. Meanwhile, another matching point pairs set B is created by the traditional key point extraction and matching algorithm. The AvgFlow estimation module judges how fast the scene motion is. Moreover, in the loss function of the optical flow network, the Euclidean distance between A and B is added as the supervisory signal.

As shown in Figure 2, we used adjacent consecutive images as input and the camera pose as output in the framework. We adopted Zhou’s [6] method, which used the occlusion mask and the flow consistency score map [27] to select the top 10% forward–backward scores in nonoccluded regions. Then, the whole system randomly selected 3k corresponding point pairs, which we denoted as A. In the KeyPoint extraction module, feature points in frames were extracted by a traditional key point extractor, such as SURF. Afterward, the feature matching method, that is, FlannMatch [28], was used to generate corresponding point pairs, which we denoted as B.

The AvgFlow estimation module thresholded the speed of the scene motion between consecutive images and chose one of the corresponding point pairs set A or B. Finally, we recovered the camera pose [R, t] by solving the essential matrix or by using the classic PnP [29] method. Normally, the camera pose is recovered by solving the essential matrix. However, when the camera has a pure rotation or a small translation, the epipolar-geometry-based method fails. Thus, in that case, we recovered the camera pose by the PnP method. Since 3D–2D point pairs are needed in the PnP resolution, we calculated the depth value of 2D points using the depth network in Monodepth2 [30]. Consequently, we could obtain the 3D point (x, y, z) from a 2D point (x, y).

### 3.2. Depth Prediction

To reduce trajectory drift, we used the depth network to provide the scale η for calculating the camera motion [R, t]. We triangulated some spatial points based on the 2D–2D corresponding point pairs and the relative pose [R, t] between two frames obtained by solving the essential matrix. Then, we aligned the pseudodepth map formed by the triangulated points with the depth map generated by the depth network to obtain the scale η. Finally, we multiplied the camera motion [R, t] obtained by the essential matrix and scale η to get the final pose motion.

Although the use of scale information provided by the depth network combined with the traditional matching method can effectively reduce trajectory drift, when the sequential frame motion is too fast, the traditional matching method can easily lose the feature points. Even if the scale η is used to assist, it still causes a trajectory drift. Therefore, when the sequential motion is too fast, it is necessary to replace the traditional matching method with an optical flow network.

Since it is difficult to acquire the ground truth for training a depth network [31,32], we used the unsupervised learning for the depth network. We mainly refer to Godard et al. [30] and joined the pose network for optimization as they do. The conversion matrix was calculated from the source frame by the pose network and the matrix converted by the pose network was projected onto the camera with the inherent internal parameter matrix to obtain the reconstructed target image. It mainly led the information of the upper layer network to the lower layer network and used the minimum reprojection error to replace the averaging error. For more details of the depth network and pose network, we recommend readers refer to Godard et al. [30].

### 3.3. Coordinate Mapping

We used the SURF+FLANN method to select corresponding point pairs as the ground truth to train the flow network without labels. The precision of the estimated trajectory was directly impacted by the accuracy of the 2D–2D or 3D–2D corresponding point pairs since our camera pose [R, t] was solved by solving the essential matrix or using the PnP method. The number of 2D–2D corresponding point pairs generated by the feature matching method was, however, lower than the number generated by the optical flow. We were unable to assign a corresponding pseudolabel to every point pair.

Thus, we used a coordinate mapping method, which took the number of point pairs generated by the traditional matching method as the baseline and projected the point pairs generated by the optical flow onto the point pairs generated by the feature matching method. To train the optical flow network in an unsupervised manner, the Euclidean distance between the point pairs uncovered by the mapping relationship was used as the error.

**Figure 3:**
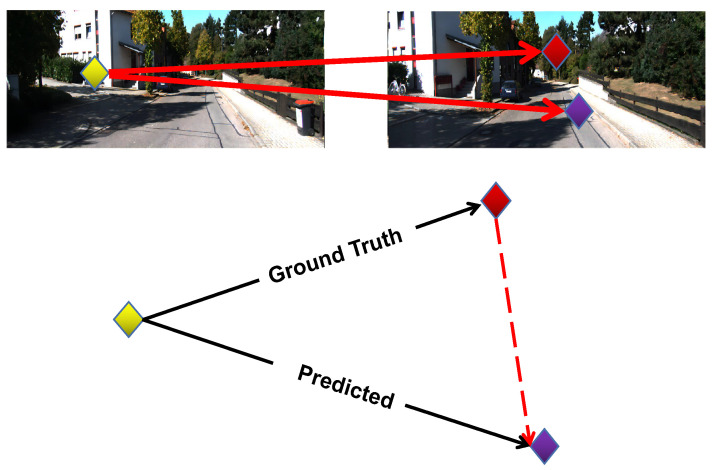
The yellow point has two corresponding points, that is, the red one and the purple one, generated by the traditional method and the flow network, respectively.

As shown in Figure 3, the yellow points are the same feature points extracted by the above two methods in the previous frame of the image, and the red points and purple points are both the corresponding points of the yellow points in the later frame of the image, where the red points were generated by the traditional matching method and the purple points were generated by the optical flow network. Ideally, the coordinates of the red and purple points should be the same and consistent. We computed the Euclidean distance between the red points and the purple points as the error to optimize the optical flow network.

**Figure 4:**
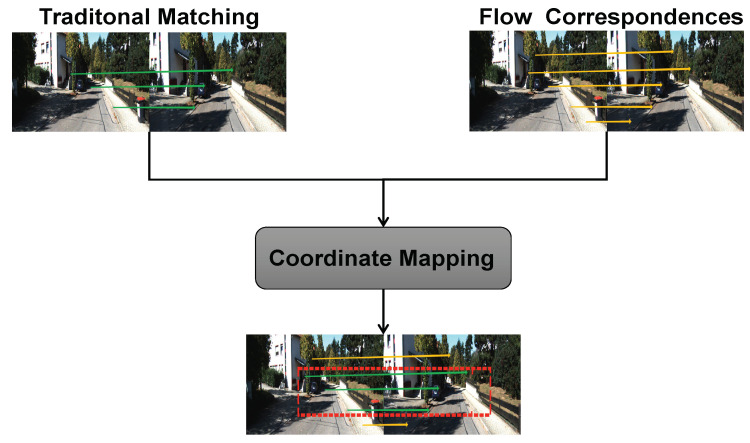
Coordinate mapping. Traditional matching is used as the benchmark, and matching point pairs are selected by coordinate mapping.

As shown in Figure 4, the red box’s corresponding point pairs indicate that the coordinates of the feature points extracted by the two methods in the previous frame are the same, but the coordinates of the feature points extracted in the later frame are different.

For example, the coordinates of the feature point ($X_{a1}$.$Y_{a1}$) extracted in the previous frame by FlowNet and the coordinates of the feature point ($X_{c1}$.$Y_{c1}$) extracted in the previous frame by the traditional matching method are exactly equal, so we consider them as the same feature point. The corresponding feature points ($X_{b1}$.$Y_{b1}$) extracted by the traditional matching method in the next frame and the corresponding feature points ($X_{d1}$.$Y_{d1}$) extracted by FlowNet in the next frame, however, are different, even though the coordinates of the feature points extracted by both methods in the previous frame are the same.

### 3.4. The Loss Function

Our depth network and optical flow network were trained independently. The total flow loss and total depth loss were defined as follows:

- Total flow loss: The total loss of our final training was L = pixel + SSIM [33] + smooth + consis + Matchloss. The pixel, SSIM, smooth and consis losses are the ones from the formula in Zhao’s paper [6]. The last one, Matchloss, is ours and is defined as follows:
  (1) $$\zeta =\sqrt{{\left(x_{2}-x_{1}\right)}^{2}+{\left(y_{2}-y_{1}\right)}^{2}}.$$
  ($x_{1}$,$y_{1}$) and ($x_{2}$,$y_{2}$) are the coordinates of the corresponding points generated by the two methods, respectively. The Euclidean distance between the corresponding point pairs generated by the two methods was added to the loss function to optimize the optical flow network.
- Total depth loss: we followed the method in Monodepth2 [30] to train the deep network. The loss of pixels was μ, which was defined as follows:
  (2) $$\mu =\left[\min_{t^{\prime }}pe\left(I_{t},I_{t^{\prime }\to t}\right)<\min_{t^{\prime }}pe\left(I_{t},I_{t^{\prime }}\right)\right],$$
  where pe is a photometric reconstruction error, $I_{t^{\prime }}$ is an unwarped source image, $I_{t^{\prime }\to t}$ is the warped image and [ ] is the Iverson bracket.

### 3.5. AvgFlow Estimation and Pose Estimation

We defined the velocity of motion between two adjacent frames as $L_{p}$ and a threshold as β. The role of the AvgFlow estimation module was to determine whether the value of $L_{p}$ was less than β. If $L_{p}$ was larger than β, then the corresponding point pairs were generated by the forward–backward flow consistency principle. Otherwise, we used the traditional matching approach to choose the corresponding point pairs. $L_{p}$ was defined as follows:

(3) $$X=x_{m}-x_{n},\;Y=y_{m}-y_{n},\;P_{i}=\left(X_{i},Y_{i}\right),$$

(4) $$\lambda a=\sum_{i=1}^{n}\sqrt{X_{i}^{2}+Y_{i}^{2}},\;\lambda b=kn,\;L_{p}=\frac{\lambda a}{\lambda b},$$

where *k* is the variable factor, ($x_{m}$,$y_{m}$) and ($x_{n}$,$y_{n}$) are pairs of matching points and $P_{i}$ is the coordinate after calculating the difference.

We used the epipolar-geometry-based method for solving the camera pose between adjacent frames given 2D–2D correspondences. F, the fundamental matrix, and E, the essential matrix, are related by the camera intrinsic K. Under normal circumstances, we recovered the camera motion [R, t] by solving F or E [34,35,36,37]. However, when the scene had a pure rotation or a small translation, the above-mentioned method failed. In that event or when $L_{p}$ was less than α, we used the PnP method to recover the camera pose. The following formulas refer to [38]:

(5) $$F=K^{-T}EK^{-1},$$

(6) $$p_{c}^{T}K^{-T}EK^{-1}p_{d}=0,$$

(7) E=[t]×R.

Here, *K* is the camera internal parameter matrix, *E* is the essential matrix and *F* is the fundamental matrix. The set of 2D–2D correspondences ($p_{c}$, $p_{d}$) can be obtained from the image pair. The *R* and t represent the motion between the two camera coordinate systems. For example, R12 and t12 indicate that the coordinates in coordinate system 1 are converted to coordinate system 2.

## 4. Experiment

### 4.1. Implementation Details

We chose the Pytorch library [39] and used Adam [40] as the optimizer. The picture size was set to 832 × 256. We set the learning rate to 0.0001 and batch size to four. The KITTI [41] odometry dataset has a total of 11 sequences with ground truth and sampled at 10 Hz. Regarding the division of the test and training sets, we followed Zhao et al. [6], Zhou et al. [19] and Bian et al. [5], using sequences 00–08 as the training set and sequences 09–10 as the testing set to train our optical flow network and depth network.

The value of β can divide the fast- and slow-motion scenes, and the larger the value of β, the lower the proportion of fast-motion scenes. The value of α is proportional to the PnP method, and the larger the value of β, the more frequently the PnP method is used. After many experimental tests, we set the value of β to 15 and the value of α to three, which gave the best accuracy and robustness for VO. For the deep network, we referred to the network structure and training method provided by Monodepth2 [30]. The deep network used ResNet18 as the encoder and the pose network was divided into a feature-coding submodule and feature-decoding submodule.

### 4.2. KeyPoint Extraction and Matching

The KeyPoint extraction module mainly used feature point extraction algorithms, including ORB, SURF and SIFT, to extract the feature points of scene images.

- ORB is the combination of the FAST feature point detection method and the BRIEF [42] feature descriptor. Although its accuracy is less than SURF and SIFT, its advantage is that its feature extraction speed is higher than that of SURF and SIFT.
- By giving values to the feature points in accordance with the local picture structure of the detected keypoints, SIFT primarily achieves rotation invariance by creating a DOG scale space. Although the extraction speed is extremely slow, its advantage is that its feature extraction accuracy is higher than that of many well-known feature extraction algorithms.
- The pyramid image created by SURF, which is an optimized version of SIFT, differs greatly from that created by SIFT. In SIFT, the picture pyramid is obtained by gradually downsampling. However, in SURF, it is mostly created by adjusting the filter’s size. Compared with SIFT, SURF is quicker.

Compared to ORB, SURF has a higher accuracy, and the computation time of SURF is lower than that of SIFT. Due to SURF’s superior accuracy and fast feature extraction speed, we primarily used SURF as the feature extractor.

Our test platform was a Core i7-9750H-CPU, NVIDIA RTX2080-GPU and Ubuntu 18.04 system. As shown in Table 1, the number of feature points extracted by SURF was much larger than that of SIFT. Although the accuracy of SURF was lower than SIFT, SURF was much faster than SIFT.

Currently, traditional matching algorithms include BF-match (brute-force matching) [43] and FLANN-match (fast nearest neighbor matching) [28]. BF-match is a classic and simple matching method. For instance, there are two sets of descriptors. One is Q = [q1, q2, …, qn], the other is K = [k1, k2, …kn]. For a random point in Q, BF-match calculates the distance from each point in K. A point pair is deemed to match if a point in K is the closest to this point in P. We stopped using BF-match since it mismatched frequently and had a sluggish matching speed.

In this paper, we chose corresponding point pairs using FLANN-match. The randomized k-d tree approach is used by FLANN-match, and two dictionary parameters must be set. FLANN-match is faster and more accurate than BF-match.

### 4.3. Performance in Normal Motion Scene

We compared our methods with deep-learning-based VO methods including SFM-Learner [19], Deep-VO-Feat [44], CC [21], SC-SfMLearner [5], TrainFlow [6], SAEVO [45] and a traditional VO method, ORB-SLAM2 [4]. Since monocular VO systems lack a scale factor, we referred to Zhao et al. [6] and applied a seven-DoF transformation to align the predicted trajectory to the ground truth. Since we combined the advantages of traditional VO methods with those of deep-learning-based VO methods, our accuracy was higher than that of pure deep-learning-based VO methods or pure traditional VO methods. Regarding the experimental settings in Table 2, only a few experimental parameters in the TrainFlow [6] method were relevant to us. The authors set the pose threshold to 10 and the maximum iteration to five. We performed several tuning tests on these parameters, and the best accuracy and robustness of VO was obtained when the pose threshold was set to five and the maximum iteration was set to one.

R (${}^{{}^{\circ}}$/100 m) and T (%) in Table 2 refer to the rotational error and the average translational error. The rotational error refers to the average rotational RMSE drift (${}^{{}^{\circ}}$/100 m) and the translational error refers to the translational root mean square error (RMSE) drift (%). As shown in Table 2, compared with TrainFlow [6], our T (%) improved by approximately 42.5% on average and R (${}^{{}^{\circ}}$/100 m) improved by approximately 11.4% on average. Our method outperformed the others by a clear margin in normal-motion scene.

### 4.4. Performance in Fast-Motion Scenes

We adopted Zhao’s approach [6] and extracted the KITTI sequences with different strides to simulate fast-motion scenes. We classified fast-motion scenes into three types, namely small-amplitude-motion scene, medium-amplitude-motion scene, and large-amplitude-motion scene.

**Figure 5:**
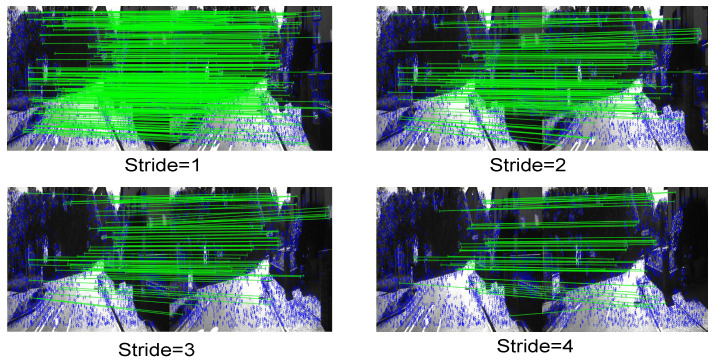
Number of matched corresponding point pairs using SURF+FLANN. As shown, when the movement gets faster (the larger the stride is), the number of found matches decreases.

The stride = 1, stride = 2, stride = 3 or stride = 4 mean we extracted a frame at 0.1 s, 0.2 s, 0.3 s or 0.4 s interval. We evaluated the robustness of the SURF+FLANN method under different strides. As shown in Figure 5, when the motion speed between adjacent frames continued to increase, the number of corresponding point pairs was constantly decreasing.

**Figure 6:**
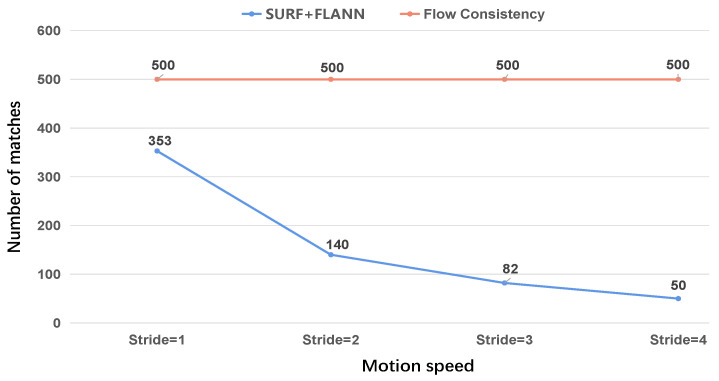
As the motion increases, the number of matches found by SURF+FLANN decreases from 353 to 50.

As shown in Figure 6, as the stride increased, the number of SURF+FLANN matches decreased from 353 to 50. However, according to the optical flow network using the principle of forward–backward flow consistency, the selected matching point pairs were dense, and the number of matches was only related to the selected threshold, independent of the stride. Therefore, when the motion between two frames was too fast, we chose to use optical flow networks to generate corresponding point pairs.

To verify the robustness of our method in fast-motion scenes, we compared it with traditional visual odometry including OBR-SLAM2 [4] and deep learning methods including SFM-Learner [19], Deep-VO-Feat [44], CC [21], SC-SfMLearner [5] and TrainFlow [6]. It was difficult to compare with some articles on fast-motion scenes. The main reasons are as follows:

- The code for some articles was not open source.
- Experimental data on fast-motion scenes were not mentioned in some articles, so we could not assert their accuracy in fast-moving scenes.
- Even with open-source code, there may be some reasons, such as incomplete code or errors in running steps, which also made it difficult to obtain their accuracy in fast-moving scenes.

### 4.5. Performance in Small-Amplitude-Motion Scene

We set the stride to two to simulate a scene with a small-amplitude motion. As shown in Table 3, the traditional visual odometry and deep learning methods, including ORB-SLAM2 [4], SFM-Learner [19], Deep-VO-Feat [44], CC [21] and SC-SfMLearner [5] all led to small trajectory drifts. Although both TrainFlow [6] and our method maintained a high accuracy, our T(%) was, on average, 40.5% higher compared to TrainFlow.

### 4.6. Performance on Medium-Amplitude-Motion Scene

We set the stride to three to simulate a scene of medium-amplitude motion. As shown in Table 4, in that scene, the trajectory of ORB-SLAM2 [4] completely lost track. Although the trajectory of deep learning methods including SFM-Learner [19], Deep-VO-Feat [44], CC [21] and SC-SfMLearner [5], did not completely lost track, it led to a trajectory drift. Since we did not rely on PoseNet to predict trajectories as TrainFlow [6] did, both achieved a better accuracy, but since we incorporated traditional matching methods, our accuracy was higher compared to TrainFlow [6], with an improvement of about 36% in T (%) and about 35% in R (${}^{{}^{\circ}}$/100 m).

### 4.7. Performance on Large-Amplitude-Motion Scene

We set the stride to four to simulate a scene of large-amplitude motion. As shown in Table 5, in that scene, the deep learning methods including SFM-Learner [19], Deep-VO-Feat [44], CC [21] and SC-SfMLearner [5] had a more serious trajectory drift than in the previous medium-amplitude-motion scene. It is clearly shown that our unsupervised system was robust and had better performance on a large-amplitude-motion scene, even compared to ORB-SLAM2, which frequently failed and lost track under fast motion. Although both TrainFlow [6] and our method maintained a high accuracy, since we incorporated traditional matching methods, our accuracy was higher compared to TrainFlow [6], with an improvement of about 21.5% in T (%) and about 4% in R (${}^{{}^{\circ}}$/100 m).

### 4.8. Ablation Study

We evaluated the impact of different factors on the TFD-VO system through detailed ablation studies. The different factors were divided into three categories, the first category was “Only Flow”, which meant we only recovered the camera pose by FlowNet and the principle of forward–backward flow consistency. The second category was “Only FPMatch”, which meant we only recovered the camera pose by the SURF+FLANN method, and the last category was “Combine”, which meant the combination of the two approaches to recover camera pose.

When the motion between two adjacent frames was too fast, the Only FPMacth method generated too few corresponding point pairs, which made it difficult to calculate the camera pose afterward and made it easy to lose track. Therefore, we replaced the traditional matching method with the principle of forward–backward flow consistency to select the corresponding point pair. Regardless of the method used to select the corresponding point pairs, the camera pose was solved by using the PnP method or solving the essential matrix, based on the previously established corresponding point pairs.

**Figure 7:**
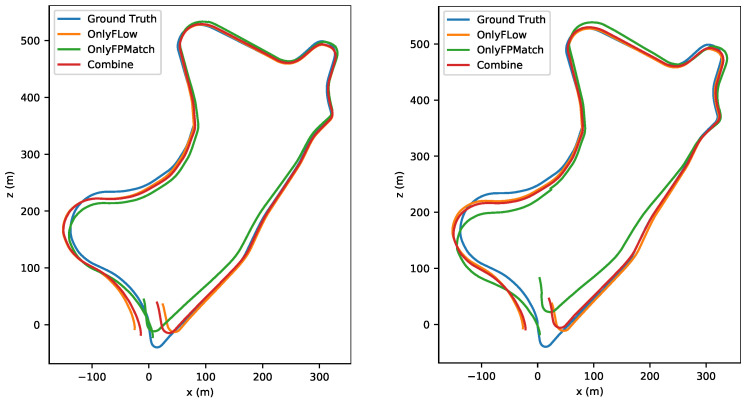
The left image shows the estimated trajectories in a normal-motion scene (stride = 1). The right image shows a small-amplitude-motion scene (stride = 2) of sequence 09 in the KITTI dataset.

**Figure 8:**
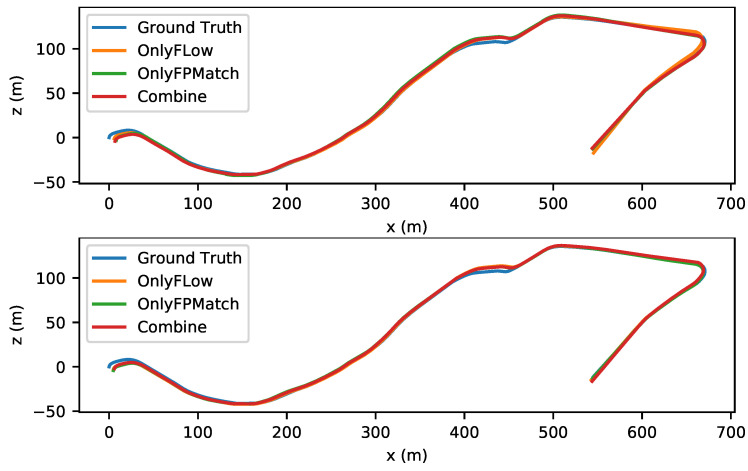
The upper image shows the estimated trajectories of a normal-motion scene (stride = 1). The lower image shows the results from a small-amplitude-motion scene (stride = 2) in sequence 10 of the KITTI dataset.

**Figure 9:**
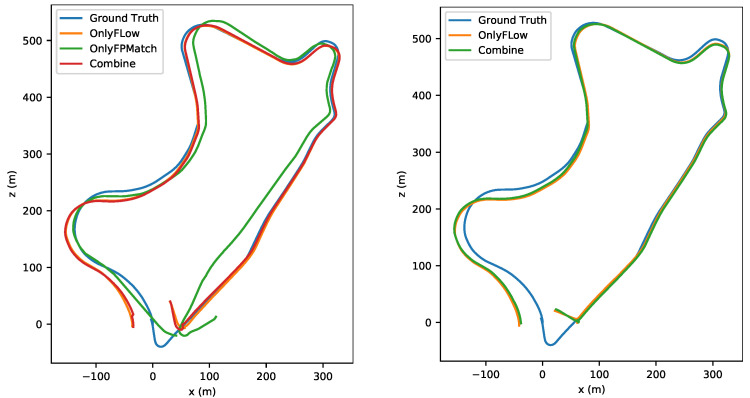
The left image shows the estimated trajectories in a medium-amplitude-motion scene (stride = 3). The right image shows the results from a large-amplitude-motion scene (stride = 4) of sequence 09 in the KITTI dataset.

**Figure 10:**
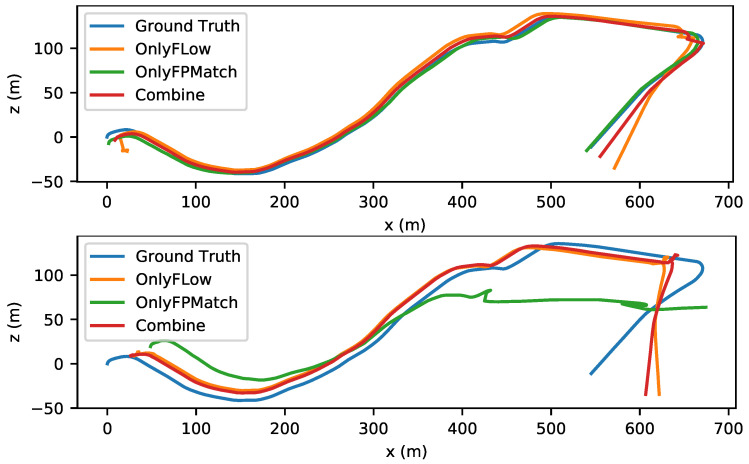
The upper image shows the estimated trajectories of a medium-amplitude-motion scene (stride = 3). The lower image shows the results from a large-amplitude-motion scene (stride = 4) in sequence 10 of the KITTI dataset.

To evaluate the effectiveness of the combination, we utilized the absolute trajectory error (ATE) as the criterion. As shown in Figure 7 and Figure 8, the trajectory accuracy was improved slightly in small-amplitude-motion scene (stride=1) and medium-amplitude-motion scene (stride = 2) after our combination. As shown in Table 6, the ATE value in any motion scenes was lowest after our combination. As shown in Figure 9 and Figure 10, when the stride was four, the feature matching method lost track in sequence 09, so no trajectory was generated, and it had a serious trajectory drift in sequence 10. As the value of the stride changed from one to two, three or four, for both Only Flow and Only FPMatch, the ATE increased. In normal-motion scene (stride = 1), Only FPMatch was preferred over Only Flow. However, in large-amplitude-motion scene (stride = 4), as shown in Figure 10, since Only FPMatch generated too few corresponding point pairs, Only Flow was preferable to Only FPMatch.

## 5. Conclusions

In this paper, we proposed an unsupervised monocular visual odometry based on the optical flow network with a feature point matching constraint. The matched feature points were used as the supervisory signal to train the optical flow network in an unsupervised manner. In contrast to supervised algorithms, our approach did not rely on ground truth labels. Moreover, to overcome the failure of feature-matching methods in fast-moving scenes, we presented an adaptive feature-matching selection module to obtain a robust pose estimation performance. Experiments demonstrated that our method had a significant improvement in the trajectory drift caused by the cumulative error and outperformed the other methods by a clear margin.

## Figures and Tables

**Table 1:** We randomly selected two adjacent images for SIFT and SURF evaluation; point A refers to the number of feature points extracted from the previous image and point B refers to the number of feature points extracted from the latter image.

	SURF	SIFT
Execution speed (ms)	**20.7**	33.8
Point A	1390	934
Point B	1413	905
Match numbers	784	495
Accuracy rate (%)	86	**88**

**Table 2:** Compared with recent works, our method produced fairly good camera pose prediction in a normal-motion scene (stride = 1). ORB-SLAM2 contained no loopback detection.

	Seq.09		Seq.10	
	T (%)	R (${}^{{}^{\circ}}$/100 m)	T (%)	R (${}^{{}^{\circ}}$/100 m)
ORB-SLAM2 [4]	9.31	0.26	2.66	**0.39**
SfM-Learner [19]	11.34	4.08	15.26	4.08
Deep-VO-Feat [44]	9.07	3.80	9.60	3.41
SAEVO [45]	8.13	2.95	6.76	2.42
CC [21]	7.71	2.32	9.87	4.47
SC-SfMLearner [5]	7.60	2.19	10.77	4.63
TrainFlow [6]	6.93	0.44	4.66	0.62
Ours	**4.29**	**0.43**	**2.46**	0.49

**Table 3:** The VO results on small-amplitude-motion scene (stride = 2). R (${}^{{}^{\circ}}$/100 m) and T (%) refer to the rotation error and the average translation error.

	Seq.09		Seq.10	
	T (%)	R (${}^{{}^{\circ}}$/100 m)	T (%)	R (${}^{{}^{\circ}}$/100 m)
ORB-SLAM2 [4]	11.12	0.33	2.97	0.36
SfM-Learner [19]	24.75	7.79	25.09	11.39
Deep-VO-Feat [44]	20.54	6.33	16.81	7.59
CC [21]	24.49	6.58	19.49	10.13
SC-SfMLearner [5]	33.35	8.21	27.21	14.04
TrainFlow [6]	7.02	**0.45**	4.94	**0.64**
Ours	**4.52**	**0.45**	**2.66**	0.68

**Table 4:** The VO results on medium-amplitude-motion scene (stride = 3). As we can see, ORB-SLAM2 was hard to initialize and lost track, while our approach outperformed the others by a clear margin.

	Seq.09		Seq.10	
	T (%)	R (${}^{{}^{\circ}}$/100 m)	T (%)	R (${}^{{}^{\circ}}$/100 m)
ORB-SLAM2 [4]	X	X	X	X
SfM-Learner [19]	49.62	13.69	33.55	16.21
Deep-VO-Feat [44]	41.24	10.80	24.17	11.31
CC [21]	41.99	11.47	30.08	14.68
SC-SfMLearner [5]	52.05	14.39	37.22	18.91
TrainFlow [6]	7.21	**0.56**	11.43	2.57
Ours	**5.36**	**0.56**	**6.21**	**1.68**

**Table 5:** The VO results on large amplitude motion scene (stride = 4). Compared with recent works, our method produced fairly good camera pose prediction in the large-amplitude-motion scene (stride = 4).

	Seq.09		Seq.10	
	T (%)	R (${}^{{}^{\circ}}$/100 m)	T (%)	R (${}^{{}^{\circ}}$/100 m)
ORB-SLAM2 [4]	X	X	X	X
SfM-Learner [19]	61.24	18.32	38.94	19.62
Deep-VO-Feat [44]	42.33	11.88	25.83	11.58
CC [21]	51.45	14.39	34.97	17.09
SC-SfMLearner [5]	59.32	17.91	42.25	21.04
TrainFlow [6]	7.72	1.14	17.30	**5.94**
Ours	**5.65**	**1.04**	**14.55**	6.24

**Table 6:** Ablation Study. “Only Flow” means only FlowNet and the principle of forward–backward flow consistency was used to recover the camera pose. “Only FPMatch” means SURF and FlANN were used to recover the camera pose. “Combine” means the combination of the two approaches was used to recover camera pose.

	Error	Only Flow	Only FPMatch	Combine
09 (stride = 1)	T error (%)	4.41	3.80	4.29
	R error (${}^{{}^{\circ}}$/100 m)	0.53	0.48	0.43
	ATE	17.19	15.02	**14.98**
09 (stride = 2)	T error (%)	4.72	6.13	4.52
	R error (${}^{{}^{\circ}}$/100 m)	0.50	1.03	0.45
	ATE	18.51	27.14	**17.95**
09 (stride = 3)	T error (%)	5.39	15.10	5.36
	R error (${}^{{}^{\circ}}$/100 m)	0.62	15.07	0.56
	ATE	22.12	30.39	**21.58**
09 (stride = 4)	T error (%)	5.83	X (track loss)	5.65
	R error (${}^{{}^{\circ}}$/100 m)	1.10	X (track loss)	1.04
	ATE	24.42	X (track loss)	**23.98**
10 (stride = 1)	T error (%)	2.83	2.89	2.46
	R error (${}^{{}^{\circ}}$/100 m)	0.65	0.63	0.49
	ATE	4.67	4.62	**4.46**
10 (stride = 2)	T error (%)	2.77	2.77	2.66
	R error (${}^{{}^{\circ}}$/100 m)	0.44	1.04	0.68
	ATE	4.72	4.93	**4.39**
10 (stride = 3)	T error (%)	5.72	4.85	6.21
	R error (${}^{{}^{\circ}}$/100 m)	1.96	2.93	1.68
	ATE	15.75	9.09	**8.87**
10 (stride = 4)	T error (%)	16.33	24.16	14.55
	R error (${}^{{}^{\circ}}$/100 m)	6.39	27.14	6.24
	ATE	34.55	65.20	**28.53**

## Data Availability

The dataset used in this paper is the publicly available KITTI dataset. It can be downloaded at the following links: https://www.cvlibs.net/datasets/kitti/index.php, accessed on 10 November 2022.
